# The ovarian estrogen synthesis function was impaired in Y123F mouse and partly restored by exogenous FSH supplement

**DOI:** 10.1186/s12958-018-0365-7

**Published:** 2018-05-04

**Authors:** Xiaoyu Tu, Miao Liu, Jianan Tang, Yu Zhang, Yan Shi, Lin Yu, Zhaogui Sun

**Affiliations:** 1 Key Lab of Reproduction Regulation of NPFPC, SIPPR, IRD, Fudan University, Xietu Road 2140, Xuhui District, Shanghai, 200032 China; 2 Women’s Hospital, School of Medicine, Zhejiang University, Hangzhou, China; 3 Key Laboratory of Contraceptive Drugs & Devices of NPFPC, Shanghai Institution of Planned Parenthood Research, Xietu Road 2140, Xuhui District, Shanghai, 200032 China

**Keywords:** Leptin receptor, Tyrosine site mutations, Infertility, Ovarian steroid hormone synthases

## Abstract

**Background:**

LepR tyrosine site mutation mice (Y123F) exhibit decreased serum E2 levels, immature reproductive organs, infertility as well as metabolic abnormalities. Although the actions of leptin and lepR in the control of reproductive function are thought to be exerted mainly via the hypothalamic-pituitary-gonadal axis, relatively less is known regarding their local effects on the peripheral ovary, especially on steroid hormone synthesis. Meanwhile, whether the decreased fertility of Y123F mouse could be restored by gonadotropin has not been clear yet.

**Methods:**

The serum levels of E2, P4, FSH, LH, T and leptin of Y123F and WT mice at the age of 12 weeks were measured by enzyme-linked immunosorbent assay. Immunohistochemistry was used to compare the distribution of hormone synthases (STAR, CYP11A1, CYP19A1, HSD17B7) and FSHR in adult mouse ovaries of two genotypes. Western blot and real-time PCR were used to detect the expression levels of four ovarian hormone synthases and JAK2-STAT3 / STAT5 signaling pathway in 4 and 12 weeks old mice, as well as the effects of exogenous hFSH stimulation on hormone synthases and FSHR.

**Results:**

Compared with WT mice, the serum levels of FSH, LH and E2 in 12-week-old Y123F mice were significantly decreased; T and leptin levels were significantly increased; but there was no significant difference of serum P4 levels. STAR, CYP11A1, HSD17B7 expression levels and the phosphorylation levels of JAK2 and STAT3 were significantly decreased in adult Y123F mice, while the expression of CYP19A1 and phospho-STAT5 were significantly increased. No significant differences were found between 4-week-old Y123F and WT mice. After exogenous hFSH stimulation, E2 levels and expression of CYP19A1 and HSD17B7 were significantly higher than that in the non-stimulated state, but significant differences still existed between Y123F and WT genotype mice under the same condition.

**Conclusions:**

Abnormal sex hormone levels of Y123F mice were due to not only decreased gonadotropin levels in the central nervous system, but also ovarian hormone synthase abnormalities in the peripheral gonads. Both FSH signaling pathway and JAK2-STAT3/STAT5 signaling pathway were involved in regulation of ovarian hormone synthases expression. Exogenous FSH just partly improved the blood E2 levels and ovarian hormone synthase expression.

## Background

Obesity has been one of the most important health problems within the world’s population; the prevalence of overweight and obesity is increasing in reproductive-aged women [1]. It has detrimental impact on female reproductive health, including ovulatory disorders [2], decreased rates of conception, infertility [3], early pregnancy loss etc. [2]. Therefore, it is of great clinical significance to study the molecular mechanisms of impaired fertility in association with obesity.

Leptin, as an important adipokine, is encoded by ob gene and secreted by adipose tissue. Leptin receptor (lepR), with multiple isoforms (lepR a, b, c, d, e, and f), was encoded by db gene [4–6]. After ligand binding, lepR activates intracellular signal transduction pathways mainly including JAK2-STAT3/STAT5 through three tyrosine residues tyr985, tyr1077 and tyr1138 [7]. Previous studies reported leptin and lepR-deficient mice, such as ob/ob or db/db mice, all displayed metabolic abnormalities and reproductive disorders [6, 8]. These fully illustrated that, in addition to controlling energy balance and body weight, leptin also served as a necessary signal to regulate female reproduction. Y123F mice are transgenic mice that are expressed mutant leptin receptors with phenylalanine (F) substitution for all three tyrosine (Y) residues [9]. Our previous study showed that Y123F female mice were infertile similar with db/db mice, including no estrous cycle, anovulation and a significant decrease in serum estradiol (E2) levels [10]. But Y123F mice’s symptoms of obesity, hyperphagia, hyperinsulinemia and impairment in glucose tolerance are less severe than db/db mice due to the remaining tyrosine-independent mechanisms. Also, Y123F mice have a longer life span than db/db mice [9]. Therefore, Y123F mice are more suitable to be a new animal model to investigate the role of leptin and lepR in reproduction, especially in obesity-related infertility.

The expression of lepR has been documented not only in the central nervous system such as hypothalamus or pituitary, but also in the peripheral gonads such as ovary granulosa or embryonic cells of several species [5, 7, 11]. However, the actions of leptin and lepR in the control of reproductive function are thought to be exerted mainly via the hypothalamic-pituitary-gonadal (HPG) axis, relatively less is known regarding their local effects on the peripheral ovary [12], especially on ovarian hormone synthesis. Therefore, in this study, we decided to observe effects of leptin and lepR on ovary itself, particularly on its steroid hormone synthesis with Y123F mouse model.

During ovarian steroidogenesis, the first step takes place within mitochondria where steroidogenic acute regulatory protein (STAR) is to facilitate the movement of cholesterol from outer mitochondrial membrane to the inner mitochondrial membrane [13]. Then, the cholesterol side-chain cleavage enzyme (P450scc or CYP11A1) converts cholesterol to pregnenolone to initiate steroidogenesis, which is a rate-limiting and quantitative regulation step. Next, most enzymes can be divided into two groups to mediate qualitative regulation determining the type of steroid to be produced: cytochrome P450 enzymes and hydroxysteroid dehydrogenases. Among them, CYP19 (P450arom) catalyzes the conversion of the C19 androgen, androstenedione and testosterone, to the C18 estrogen, estrone and estradiol respectively. HSD17B7 primarily activates estrone to estradiol, especially in the luteal phase of the rodent ovarian cycle, which is responsible for the final step in estradiol synthesis [13, 14]. In our previous study, we verified an elevated expression of site-mutated lepR in Y123F mouse ovaries and showed some accompanied gene profiling changes, particularly including steroid hormone biosynthesis enzyme genes such as Hsd17b7 or Star [10]. So far there has been no study regarding the relation between lepR and steroidogenic enzymes as well as involved mechanism. Accordingly, in this study, we evaluate sex hormone levels in adult Y123F female mice compared with WT mice; then we focus on Y123F mice ovaries to investigate changes of steroidogenic enzymes and JAK2-STAT3/STAT5 signaling pathway. Finally, we explore whether the decreased fertility of Y123F mouse could be restored by exogenous hFSH supplement.

## Methods

### Transgenic mice

Y123F mice, a knock-in line of mice that expressed mutant leptin receptors with phenylalanine substitution for all three tyrosines, were generated by Liu Yong, et al. [9]. Heterozygous Y123F mice were kindly provided by Dr. Shi Huijuan at Shanghai Institute of Planned Parenthood Research, Shanghai, China. They were backcrossed to C57BL/6 mice (purchased from Shanghai Laboratory Animal Research Center, Shanghai, China) for at least three generations to yield heterozygous Y123F mice in the C57BL/6 background, then heterozygous Y123F mice inbred to produce homozygous Y123F mice and WT littermates as described previously [10]. The mice were genotyped by PCR analysis of tail-tip DNA at 4 weeks of age with primers: F: CTA CTG GAA TGG AAC CTT GAG GCT TC; R: CAT TTT TCC TTT TAT CAG ACC AGC AAC C. All mice were housed in standard plastic rodent cages and maintained in a temperature-and light-controlled room (22–24 °C with 55–65% relative humidity and a 12/12 h light/dark cycle) with ad libitum feed and water. The study was approved by the Animal Ethics Committee at Shanghai Institute of Planned Parenthood Research. Animals were cared for in accordance with the guidelines of Experimental Animals.

### Sample collection and hormone test

At the 4th week and 12th week after birth, mice were euthanized by cervical dislocation, ovaries were excised, cleaned of fat, weighed, and frozen at − 80 °C. At 12 weeks of age, blood samples were collected and centrifuged, and the serum was stored at − 20 °C for further analysis. 12-week-old female mice were divided into homozygous Y123F group and WT group. Mice in each group were randomly administered an intraperitoneal injection of 10 IU human follicle stimulating hormone (hFSH, gonal-f, Merck Serono Co., Ltd.) or equal does of phosphate buffer solution (PBS). After 48 h, they were sacrificed to collect ovaries and serum as above. All WT mice that were chosen as the control were at the stage of metestrus or diestrus. Serum hormone levels of progesterone (P4) and E2 were determined with Roche Cobas e601 electrochemical luminescence immunity analyzer (Roche, Switzerland) and were carried out in the Clinical Laboratory of ShangHai Institute of Planned Parenthood Research Hospital. Leptin, follicle stimulating hormone (FSH) and luteinizing hormone (LH) were measured by commercial ELISA kits (Elabscience Biotechnology Co., Ltd., Wuhan, China) according to the manufacturer’s instructions. Testosterone (T) was measured by commercial ELISA kit (R&D, USA) according to the manufacturer’s instructions. Intra-assay and interassay coefficients of variation were both < 10%.

### Immunohistochemistry

To determine the cellular localization of steroidogenic enzymes and related receptors in the ovary, immunohistochemistry (IHC) were performed as described previously [10, 15]. Mouse ovaries were randomly collected from 12-week-old female mice of WT and Y123F groups (WT mice, *n* = 3; Y123F mice, n = 3). In brief, ovarian tissue was embedded by paraffin. Sections of paraffin-embedded ovaries (5 μm) were collected on glass slides, then deparaffinized and hydrated. Antigen retrieval was achieved by microwaving the slides in 0.01 M sodium citrate solution (pH 6.0) for 15 min, cooling to room temperature and washing three times in phosphate buffered saline (PBS) each for 5 min. After washing, the slides were blocked with 3% hydrogen peroxide in PBS for 10 min to quench endogenous peroxidase activity. The nonspecific background was eliminated by blocking with 10% lamb serum in PBS for 1 h at room temperature. The slides were then incubated with the primary antibody at 4 °C overnight. The primary antibodies included STAR (1:100 dilution, Proteintech Group, USA), CYP11A1 (1:100 dilution, Proteintech Group, USA), CYP19A1 (1:100 dilution, Proteintech Group, USA), HSD17B7 (1:100 dilution, Proteintech Group, USA), and FSHR (1:200 dilution, Abways, China). The next day, after washing with PBS, the slides were incubated for 1 h at room temperature with biotinylated goat anti-rabbit IgG secondary antibody (1:200 dilution, Invitrogen, USA). The antibody dilution buffer was 10% lamb serum in PBS. Immunoreactive signals were detected using streptavidin-HRP and VECTOR Nova RED Peroxidase (HRP) Substrate Kit (Vectorlabs, Burlingame, USA) at room temperature. Slides were counterstained with hematoxylin. A negative control was performed without the primary antibody incubation step. Immunostaining was observed using a Nikon Eclipse 50i microscope (Nikon, Tokyo, Japan) and captured by NIS-Element F Software. Every antibody was repeated in slides from three separate ovaries with identical genotype.

### Protein extraction and western blot

Total protein was extracted from mixed ovaries (WT mice, *n* = 5; Y123F mice, n = 5) as described earlier [10]. In brief, ovary tissues were harvested and lysed in lysis buffer adding with 1 mM phenylmethylsulphonyl fluoride on ice (Beyotime, Shanghai, China). Lysates were homogenized using a homogenizer and centrifuged at 12000 g for 15 min at 4 °C and the protein concentrations were measured using a BCA Protein Assay Kit (CWBIO, Beijing, China). A total of 30 μg protein was boiled in 1× loading buffer to denature, then added to each lane and separated by 10% SDS-PAGE for 1.5 h. The bands were transferred to a 0.45-μm nitrocellulose membrane (ImmobilionTM-NC Millipore Corporation, USA) for 1.5~ 2 h. The membranes were then blocked with 7% nonfat dry milk powder (NFDMP) in Tris-buffered saline (TBS: 2 mM Tris-HCl, 15 mM NaCl, pH 7.4), containing 0.1% Tween-20 (TBST) for 2 h at room temperature. Then they were incubated with the primary antibody at 4 °C overnight. The primary antibodies included STAR, CYP11A1, CYP19A1, HSD17B7 (1:1000 dilution, Proteintech Group, USA), JAK2, Phospho-JAK2 (Tyr1007/1008) (1:1000 dilution, Cell Signaling Technology, USA), STAT3, Phospho-STAT3 (Tyr705), STAT5A/B, Phospho-STAT5A (Tyr694) and FSHR (1:1000 dilution, Abways, China). Beta Actin antibody (1:5000 dilution, Proteintech Group, USA) was used as the internal control. These antibodies were diluted in TBST with 3.5% NFDMP. The next day, the membranes were washed three times in TBST each for 5 min and further incubated with HRP conjugated anti-rabbit or anti-mouse secondary antibodies (1:5000 dilution, Proteintech Group, USA) for 1 h at room temperature. After washing again in TBST, the signal was detected by Tanon™ High-sig ECL Western Blotting Substrate (Tanon, China) and analyzed using the FluorChem Gel documentation system (Tanon, China). The results were obtained from three independent repeated experiments with three pools of mixed ovary protein.

### RNA extraction and quantitative real-time PCR (qPCR)

QPCR was performed to quantify the expressions of *Star, Cyp19A1, Cyp11A1, Hsd17b7, Fshr, Jak2, Stat3* and *Stat5* in the ovary. Expression levels were normalized against *Gapdh* expression. The primer sets are shown in Table 1. Total RNA was extracted from mixed ovaries (WT mice, *n* = 3; Y123F mice, *n* = 3) using Trizol (Invitrogen, USA) according to the manufacturer’s instructions. RNA quantity and purity was confirmed by NanoDrop ASP-3700 spectrophotometer (ACTGene, Piscataway, NJ, USA), OD 260/280 nm ratios between 1.8 and 2.0 were obtained for all RNA samples. Total RNA from each sample was reverse transcribed into cDNA using a standard oligo-dT RT protocol with a ReverTra Ace qPCR RT Master Mix with gDNA Remover Kit (Toyobo, Osaka, Japan) according to the manufacturer’s instructions. QPCR reactions were performed on an Illumina-Eco System (Illumina, USA) in a 10μl reaction volume with SYBR Green I Master Mix reagent (Toyobo, Osaka, Japan) and 5 pmol of the specific primer pairs shown in Table 1. The PCR thermal cycling conditions were 95 °C for 10 min for polymerase activation and the initial denaturation step, followed by 40 cycles with denaturation at 95 °C for 30s, annealing at 60 °C for 30s and extension at 72 °C for 30s. A melting curve analysis was recorded at the end of the amplification to evaluate the absence of contaminants or primer dimers. Each set of qPCR reactions was repeated three times, and the levels of gene expression were expressed in the form of 2^-ΔΔCt^. The results were repeated in three pools of total RNA from mixed ovaries.

**Table 1:** Primers used in quantitative real-time PCR

Gene	Accession number	Primer sequences (5′–3′)
*Star*	NM_011485.4	Upstream: CGGGTCTTGGGTCCTGGATTGT
		Downstream: GCTGCCCTCGCTCACCTTAAG
*Cyp19a1*	NM_007810.3	Upstream: TCGTCGCAGAGTATCCAGAGGT
		Downstream: CGCATGACCAAGTCCACAACAG
*Cyp11a1*	NM_019779.3	Upstream: GCTCAACCTGCCTCCAGACTTC
		Downstream: CCTCCTGCCAGCATCTCGGTAA
*Hsd17b7*	NM _010476.3	Upstream: CTGTGACACCGTACAACGGA
		Downstream: GCTCGGGTGATCCGATTTCT
*Jak2*	NM_008413.3	Upstream: AGATGGGAAGGGAAGGGTGTGG
		Downstream: AAGAGGGAGCAGCACGAAGGAT
*Stat3*	NM_213659.3	Upstream: TCGGTTGGAGGTGTGAGGTAGG
		Downstream: GTGGGAAAGGAAGGCAGGTTGA
*Stat5*	NM_001164062.1	Upstream: CAGAGTCGGTGACGGAGGAGAA
		Downstream: GCACGGTGGCAGTAGCATTGT
*Gapdh*	NM _001289726.1	Upstream: GTGAAGGTCGGTGTGAACGGATT
		Downstream: GGTCTCGCTCCTGGAAGATGGT

### Statistical analysis

All data are presented as Mean ± SD or Mean ± SEM as indicated. The statistical analyses and data graphing were conducted using the GraphPad Prism 5 software. WB band intensities were analyzed by Image J software. The statistical analyses were performed with unpaired two-tailed Student’s t test; a *p*-value < 0.05 was considered statistically significant.

## Results

### Y123F mice exhibited disordered sex hormone secretion

**Fig. 1:**
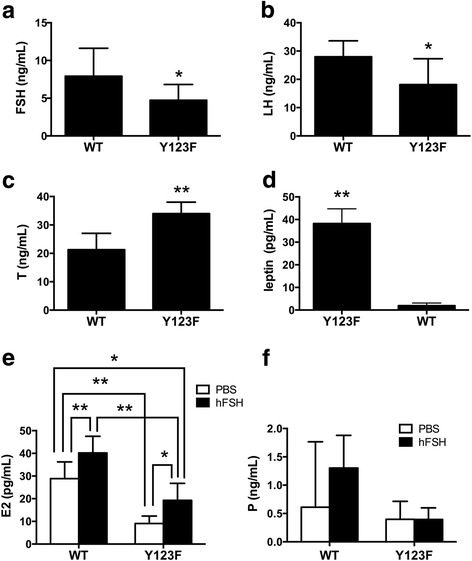
The hormone determination in wild type (WT) and Y123F homozygous (Y123F) mice. The FSH, LH, T, E2, P and leptin levels in wild type and Y123F homozygous mouse blood were determined. **a** The blood FSH levels in WT (*n* = 8) were significantly higher than Y123F (*n* = 9) mice (*p* < 0.05). **b** The blood LH levels in WT (*n* = 7) were significantly higher than Y123F (*n* = 9) mice (*p* < 0.05). **c** The blood T levels in WT (*n* = 5) were significantly lower than Y123F (n = 5) mice (*p* < 0.01). **d** The blood leptin levels in WT (*n* = 7) were significantly lower than Y123F (n = 9) mice (*p* < 0.01). **e** The blood E2 levels in WT (*n* = 9/13) and Y123F (*n* = 7/6) mice after an intraperitoneal injection of 10 IU hFSH or PBS. **f** The blood P levels in WT (*n* = 5/8) and Y123F (*n* = 9/6) mice after an intraperitoneal injection of 10 IU hFSH or PBS. Data are expressed as the mean ± SD, *p < 0.05, **p < 0.01 indicate a significant difference between the WT and Y123F groups

In view of the uncertainty of mouse breeding, the difficulty of blood collection, the estrous cycle of WT mice and the limited volume of blood samples, the final sample size of each indicator in both groups is not necessarily equal. The results showed that serum FSH of Y123F mice (*n* = 9) were lower than that of WT mice (*n* = 8) (*p* < 0.05) (Fig. 1a). Serum LH levels of Y123F mice (*n* = 9) were lower than that of WT mice (*n* = 7) (*p* < 0.05) (Fig. 1b). T levels were significantly higher in Y123F mice (*n* = 5) (*p* < 0.01) than that of WT mice (*n* = 5) (*p* < 0.01) (Fig. 1c). Leptin levels were significantly higher in Y123F mice (*n* = 9) than that of WT mice (*n* = 7) (*p* < 0.01) (Fig. 1d).

### Steroidogenic enzymes changed in adult Y123F mouse ovaries compared with WT mouse ovaries

**Fig. 2:**
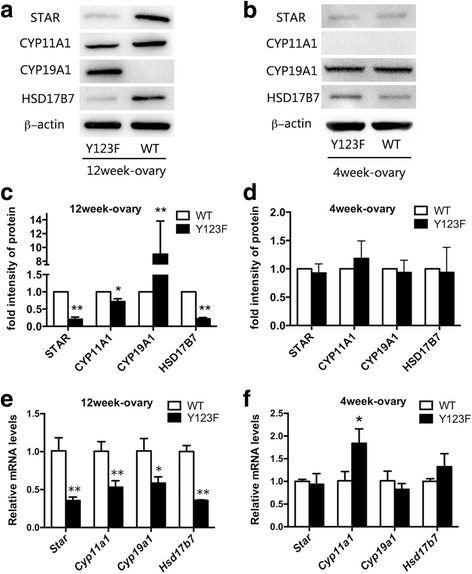
Comparison of ovary steroidogenic enzyme protein and mRNA levels of WT and Y123F mouse ovaries at the age of 4 weeks and 12 weeks. **a** Western blotting analysis of ovary steroidogenic enzyme expressions in WT and Y123F mouse ovaries at the age of 12 weeks. **c** Densitometric analysis of Western blotting detection of ovary steroidogenic enzyme expressions in WT and Y123F mouse ovaries at the age of 12 weeks. **e** QPCR analysis of ovary steroidogenic enzyme relative mRNA expressions levels in WT and Y123F mouse ovaries at the age of 12 weeks. **b** Western blotting analysis of ovary steroidogenic enzyme expressions in WT and Y123F mouse ovaries at the age of 4 weeks. **d** Densitometric analysis of Western blotting detection of ovary steroidogenic enzyme expressions in WT and Y123F mouse ovaries at the age of 4 weeks. **f** QPCR analysis of ovary steroidogenic enzyme relative mRNA expressions levels in WT and Y123F mouse ovaries at the age of 4 weeks. Data of WB results are expressed as the mean ± SD of three independent experiments. Data of QPCR results are expressed as the mean ± SEM of three independent experiments, *p < 0.05, **p < 0.01 indicate a significant difference between the WT and Y123F groups

**Fig. 3:**
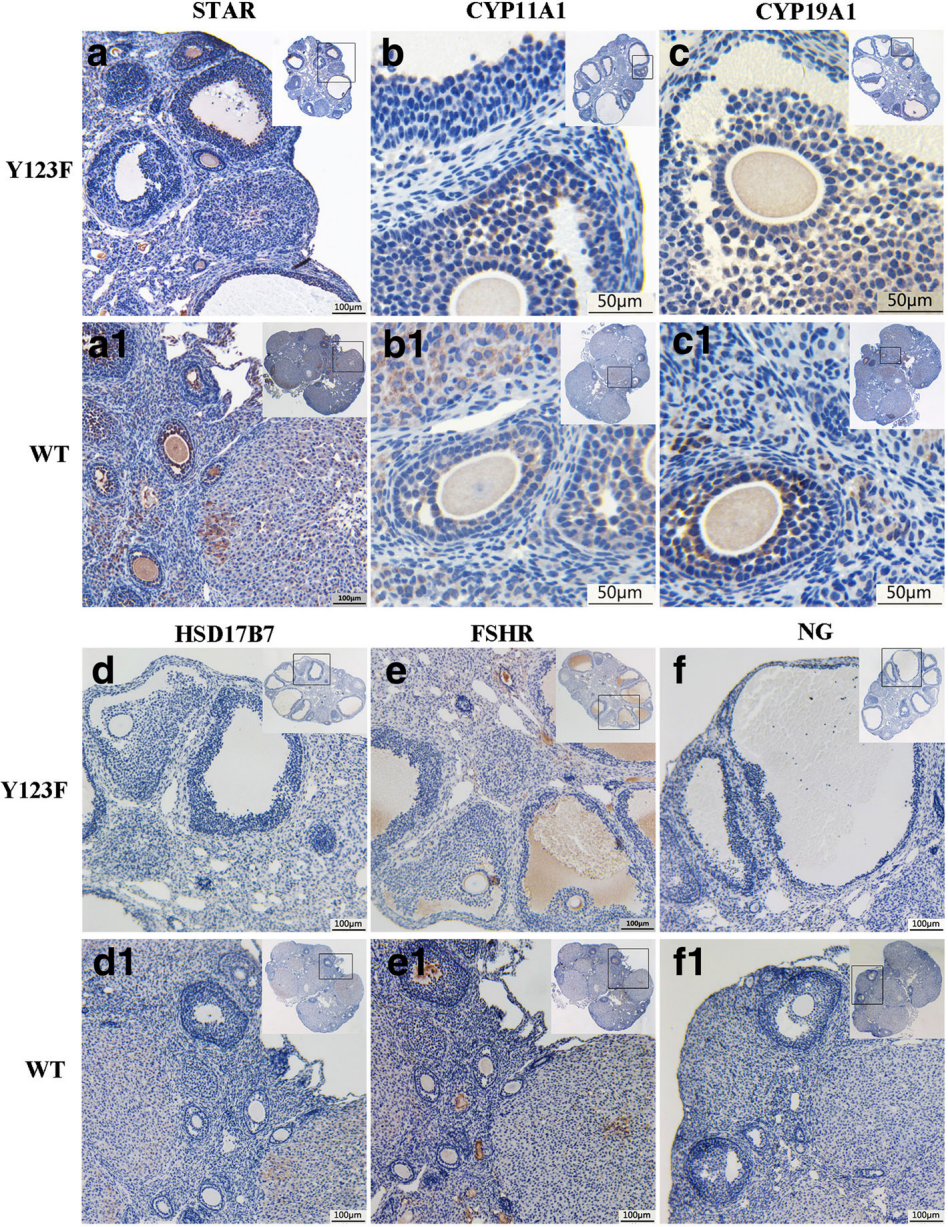
Immunohistochemical detection of ovary steroidogenic enzyme expressions in 12-week-old Y123F and WT mouse ovaries. (A-A1) The 10 × 10 magnification immunocytochemistry images of STAR expression as indicated by the dotted squares in Y123F mouse ovaries (**a**) and WT mouse ovaries (A1). (B-B1) The 40 × 10 magnification immunocytochemistry images of CYP11A1 expression as indicated by the dotted squares in Y123F mouse ovaries (**b**) and WT mouse ovaries (B1). (C-C1) The 40 × 10 magnification immunocytochemistry images of CYP19A1 expression as indicated by the dotted squares in Y123F mouse ovaries (**c**) and WT mouse ovaries (C1). (D-D1) The 10 × 10 magnification immunocytochemistry images of HSD17B7 expression as indicated by the dotted squares in Y123F mouse ovaries (**d**) and WT mouse ovaries (D1). (E-E1) The 10 × 10 magnification immunocytochemistry images of FSHR expression as indicated by the dotted squares in Y123F mouse ovaries (**e**) and WT mouse ovaries (E1). (F-F1) The 10 × 10 magnification immunocytochemistry images of negative control as indicated by the dotted squares in Y123F mouse ovaries (**f**) and WT mouse ovaries (F1)

According to cDNA Microarray results of Y123F ovaries in our previous research [10], we decided to investigate the expression of four ovary steroidogenic enzymes in 12-week-old Y123F ovaries: STAR, CYP11A1, CYP19A1, HSD17B7. WB results showed that STAR, CYP11A1 and HSD17B7 expressions were significantly decreased (*p* < 0.05); while CYP19A1 expressions were increased (*p* < 0.01) in adult Y123F mouse ovaries compared with WT ovaries (Fig. 2a, c). Meanwhile, relative mRNA expression levels of these genes were all dramatically decreased in adult Y123F mouse ovaries (*p* < 0.05) (Fig. 2e). However, comparing these steroidogenic enzymes expression changes of immature mouse ovaries (4-week-old) in both groups, only relative mRNA expression levels of CYP11A1 were higher in Y123F mouse ovaries than that of WT ovaries (*p* < 0.01). Apart from that, there was nearly no difference of four steroidogenic enzyme expressions between two groups through WB and qRT-PCR tests (*p* > 0.05) (Fig. 2b, d, f). The contradictory results of CYP19A1 protein and mRNA levels in Y123F mouse ovaries gave the impetus for us to examine these enzymes distribution and expression in ovaries through IHC. The IHC results showed that CYP19A1 mainly located in cumulus granulosa cells of tertiary follicles in Y123F mouse ovaries while in the granulosa cells of secondary follicle and corpus luteum in WT mouse ovaries (Fig. 3c, C1). Meanwhile, STAR widely distributed in the whole WT ovaries but only in some granulosa cells of Y123F ovaries (Fig. 3a, A1). CYP11A1 had the similar distribution with CYP19A1 in mouse ovaries of both groups (Fig. 3b, B1). HSD17B7 demonstrated fewer expressions in the whole Y123F mouse ovary than WT ovary where most of the corpora lutea were positive (Fig. 3d, D1). As a whole, these results indicated that lepR deficiency had a negative influence on steroidogenic enzymes production in adult Y123F mouse ovaries.

### LepR deficiency affected JAK2-STAT3/STAT5 signaling pathway in ovaries

**Fig. 4:**
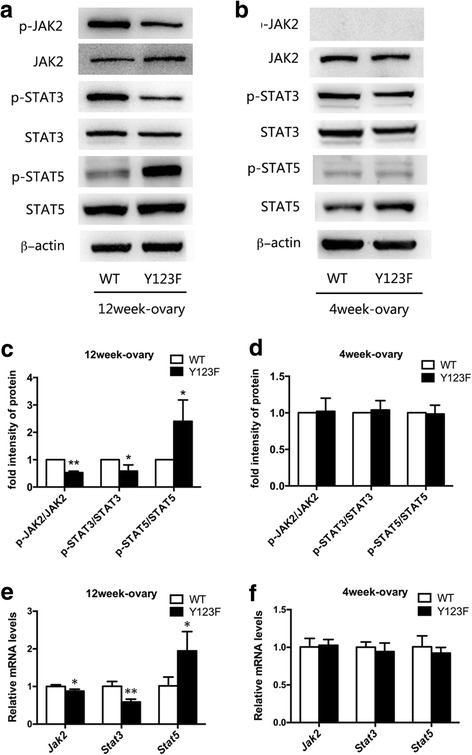
Detections of JAK2-STAT3/STAT5 signaling pathway of WT and Y123F mouse ovaries at the age of 4 weeks and 12 weeks. **a** Western blotting analysis of phospho-JAK2, JAK2, phospho-STAT3, STAT3, phospho-STAT5, STAT5 expressions in WT and Y123F mouse ovaries at the age of 12 weeks. **c** Densitometric analysis of Western blotting detection of phospho-JAK2, phospho-STAT3 and phospho-STAT5 levels in WT and Y123F mouse ovaries at the age of 12 weeks. **e** QPCR analysis of *Jak2, Stat3* and *Stat5* relative mRNA expressions levels in WT and Y123F mouse ovaries at the age of 12 weeks. **b** Western blotting analysis of phospho-JAK2, JAK2, phospho-STAT3, STAT3, phospho-STAT5, STAT5 expressions in WT and Y123F mouse ovaries at the age of 4 weeks. **d** Densitometric analysis of Western blotting detection of phospho-JAK2, phospho-STAT3 and phospho-STAT5 levels in WT and Y123F mouse ovaries at the age of 4 weeks. **f** QPCR analysis of *Jak2, Stat3* and *Stat5* relative mRNA expressions levels in WT and Y123F mouse ovaries at the age of 4 weeks. Data of WB results are expressed as the mean ± SD of three independent experiments. Data of QPCR results are expressed as the mean ± SEM of three independent experiments, *p < 0.05, **p < 0.01 indicate a significant difference between the WT and Y123F groups

As a lepR tyrosine site mutations transgenic mouse model, whether the JAK2-STAT3/STAT5 signaling pathway changed in local ovaries of Y123F mice needed to be determined. So we compared the levels of phospho-JAK2, JAK2, phospho-STAT3, STAT3, phospho-STAT5, STAT5 between Y123F and WT mouse ovaries. All determinations were performed in 4-week-old and 12-week-old mice respectively, and we found no significant differences in WB and qRT-PCR results at the age of 4 weeks (*p* > 0.05) (Fig. 4b, d, f). However, in 12-week-old ovaries, WB and qRT-PCR results showed that phosphorylation of JAK2 and STAT3 markedly decreased while phosphorylation of STAT5 dramatically increased in Y123F ovaries (*p* < 0.05) (Fig. 4a, c, e).

### Exogenous hFSH partly restored serum E2 levels and ovary steroidogenic enzymes expression of Y123F mouse

**Fig. 5:**
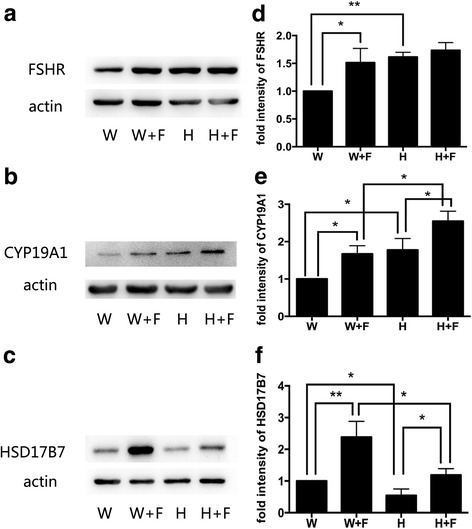
Detections of FSHR, CYP19A1, HSD17B7 expression changes in WT and Y123F mouse ovaries after injection of hFSH or PBS at the age of 12 weeks. **a** FSHR expression changes in WT mouse ovaries after injection of PBS (W) or hFSH (W + F) and in Y123F mouse ovaries after injection of PBS (H) or hFSH (H + F). **b** CYP19A1 expression changes in WT mouse ovaries after injection of PBS (W) or hFSH (W + F) and in Y123F mouse ovaries after injection of PBS (H) or hFSH (H + F). **c** HSD17B7 expression changes in WT mouse ovaries after injection of PBS (W) or hFSH (W + F) and in Y123F mouse ovaries after injection of PBS (H) or hFSH (H + F). **d-f** Densitometric analysis of Western blotting detection of FSHR (**d**), CYP19A1 (**e**) and HSD17B7 (**f**) expression changes in WT and Y123F mouse ovaries after injection of hFSH or PBS. Data are expressed as the mean ± SD of three independent experiments, *p < 0.05, **p < 0.01 indicate a significant difference between the WT and Y123F groups

Since serum E2 and FSH levels were decreased in Y123F mice, we considered whether supplement of exogenous hFSH could improve ovary function. With PBS as a control, we found that E2 levels were increased after hFSH stimulation in both genotypes, for Y123F mice (*p* < 0.05), but for WT mice (*p* < 0.01). When comparing between genotypes, for PBS injection mice, E2 levels were higher in WT mice (*n* = 13) than Y123F mice (*n* = 6) (*p* < 0.01); for hFSH injection mice, E2 levels were higher in WT mice (*n* = 9) than Y123F mice (*n* = 7) (*p* < 0.01). E2 levels in Y123F mice with hFSH injection (*n* = 7) were still lower than that in WT mice with PBS injection (*n* = 13) (*p* < 0.05) (Fig. 1e). However, there was no significant difference of serum P4 levels after hFSH injection in both genotypes (*p* > 0.05) (Fig. 1f). At the same time, we found that only CYP19A1 increased responding to hFSH induction in Y123F ovaries, which was consistent with that in WT mice (*p* < 0.05) (Fig. 5b, e). HSD17B7 just had a slight increase in Y123F ovaries after hFSH injection (*p* < 0.05) (Fig. 5c, f). Surprisingly, Y123F mouse ovaries had higher expression of FSHR than WT ovaries, but FSHR in Y123F ovaries showed no significant changes after stimulation (Fig. 5a, d). So we further detected FSHR distribution in ovaries of both groups and found that positive FSHR staining mostly located in follicular cavities and rarely in corpus luteum by IHC (Fig. 3e, E1), which contributed to the raised expression in Y123F mouse ovaries detected by WB.

## Discussion

Observational studies from humans or animal models with leptin or leptin-receptor deficiency have fully indicated that leptin-lepR system are involved in maintaining normal reproductive function [6, 16–18]. Although the central effects of leptin on hypothalamic and pituitary function have been examined extensively [19, 20], the contribution of direct leptin actions on the ovary in obesity-related infertility/subfertility remains incompletely understood. In this study, using the Y123F mouse model, we mainly explored the effects of lepR tyrosine site mutations on serum estrogen levels and local ovary steroidogenic enzymes changes. The novel finding of direct actions of leptin on the ovary may account for some adverse effects of obesity on ovarian function.

Firstly, according to our previous study that Y123F mice had low E2 level, no estrous cycle and anovulation [10], we fully tested sex hormone levels of Y123F mice at the age of 12 weeks and found that the hypothalamic hypogonadism existed. In accordance with this result, previous studies demonstrated that leptin null (ob/ob) and leptin receptor null (db/db) mice exhibited low gonadotrophin concentrations, immature reproductive organs, and impaired sexual maturation [21]. Also, leptin and leptin receptor gene defects result in human obesity and delayed puberty, oligo-anovulation, or subfertility in most human populations [22]. Since leptin receptor is expressed abundantly within the hypothalamus and pituitary [7, 23, 24], Watanobe*,* et al. showed that leptin acted within the hypothalamus of animals to stimulate the release of GnRH within the median eminence-arcuate nucleus in fasted animals [25]. Nicole Bellefontaine*,* et al. reported that leptin plays a central role in regulating the HPG axis to increase circulating LH levels in vivo through the activation of neuronal NO synthase in neurons of the preoptic region [26], in addition to through neuropeptides such as NPY [27] or kisspeptin [28]. Wen H. Yu*,* et al. also confirmed that, in vitro pituitary tissue culture; leptin induces a dose-related increase in LH, FSH, and prolactin release [20] via nitric oxide synthase activation in the gonadotropes [29]. Female fertility relies on the regulation of sex hormones and positive/ negative feedback exists in HPG axis [30, 31]. Due to the direct stimulatory effects of leptin on the HPG axis, thus for Y123F mice, abnormal lepR in central nervous systems resulted in low gonadotropin secretion, subsequently contributing to the low E2 levels.

Considering clinical patients undergoing assisted reproductive technology (ART), classic controlled ovarian hyperstimulation was performed using GnRH-agonist long protocol with exogenous recombinant FSH to stimulate oocyte development as well as increase E2 levels [2, 32]. We tried to elevate E2 levels in Y123F mice by supplementing exogenous hFSH. Surprisingly, low serum E2 levels were only partly restored due to the active response of the ovary to FSH, still remained a significant lower level compared with WT mice. So we hypothesized that low E2 levels of Y123F mouse were not only due to low gonadotropin levels, but also probably due to the local factors in the ovary directly relating to leptin and lepR, such as steroidogenisis disorder,

It is well established that a series of ovary steroidogenic enzymes modulate hormone biosynthesis, which subsequently regulates reproductive functions such as follicle development and ovulation [13]. Here, we found that ovary steroidogenic enzymes changed in 12-week-old Y123F mice, with a remarkable decrease of STAR, CYP11A1, HSD17B7, as well as an increase of CYP19A1, fully confirmed our hypothesis. Several studies have reported direct local leptin effects on ovarian functions. Zachow RJ*,* et al. indicated that leptin antagonizes the stimulatory effects of transforming growth factor-β and insulin-like growth factor-I on FSH-dependent estrogen production by a mechanism involving the leptin-induced attenuation of P450arom activity and mRNA expression in rat ovarian granulosa cells (GC) in vitro [33, 34]. Ghizzoni L*,* et al. found that 30% decrease in E2 production was caused by the certain Leptin concentration; while P4 levels were not influenced in the culture media of human granulosa-lutein cells obtained from follicular fluid of women undergoing in vitro fertilization. They concluded that leptin suppresses E2 secretion by interfering with either the translational or post-translational steps of the baseline CYP17 and/or aromatase synthesis and/or the activation of the enzymes [35]. In other species, such as sheep or bovine, leptin also inflicted a negative effect on ovarian steroidogenesis in vitro and in vivo experiments, which were corresponding with our results [36, 37]. However, some studies showed stimulatory effects of leptin on the ovary [38, 39]. These discrepancies may stem from differences in the doses of leptin and species as well as experiment design. In our study, ovary steroidogenic enzymes expressed abnormally in local ovary of lepR-deficient Y123F mice, even if to supplement exogenous hFSH, the expression defects of ovary steroidogenic enzymes were just partly recovered. But WB results showed that FSHR had higher expression in Y123F mice than WT mice, which had no significant increase after hFSH stimulation. By means of IHC, we found that higher expression of FSHR overall in Y123F mouse ovaries dectected by WB was due to more antral follicles in Y123F ovaries than in WT ovaries. But its feedback regulation may be up to saturation, leading to limited improvement of ovary steroidgenesis via hFSH supplement. Overall, our results fully illustrated that leptin and lepR in the Y123F mouse ovary had a negative effect on local ovarian steroidogenesis, independently of its central actions in the hypothalamus and pituitary.

Apart from previous studies above, Clark BJ and Lin D, et al. confirmed that overexpression of mouse STAR in mouse leydig MA-10 cells increased their basal steroidogenic rate [40], and co-transfection of expression vectors for both StAR and the P450scc system in nonsteroidogenic COS-1 cells augmented pregnenolone synthesis more than that obtained with the P450scc system alone [41]. Thus, in this study, the insufficiency of STAR, CYP11A1 and HSD17B7 can directly disrupt estradiol biosynthesis, leading to the low E2 levels of Y123F mice. It’s worth mentioning that Y123F mouse ovaries had higher expression of CYP19A1 and higher level of testosterone. As the substrate of estrogen biosynthesis, we considered that testosterone induced a compensatory response of higher expression of CYP19A1 to facilitate more conversion from testosterone to estradiol [13]. But IHC showed that CYP19A1 mainly distributed in cumulus granulosa cells of tertiary follicles of Y123F mouse ovaries; and while mostly in the granulosa cells of secondary follicle and corpora lutea of WT mouse ovaries. So we supposed that estradiol produced in granulosa cells of Y123F mouse ovaries could be less available to get into the blood circulation due to the lack of corpus luteum that had enriching blood vessels, ultimately resulting in low blood E2 levels.

Intriguingly, there was no significant difference in all 4-week-old mice. These findings suggested that lepR tyrosine site mutations led to ovary steroidogenic enzymes changes only in adult mice, which had correlations with metabolic abnormalities due to fatness. Before 4 weeks, on one hand, mice did not reach up to sexual maturity and most of steroidogenic enzymes remained inactive; on the other hand, Y123F mice still maintained regular body weight due to complete compensation for lepR deficiency by other signal pathway [9]. Corresponding with our finding, Luba Sominsky and colleagues have shown that neonatal overfeeding increased circulating levels of leptin and reduced the number of ovarian follicles, specifically, the primordial follicle pool in adult rats. The adult rats had increased levels of ovarian leptin and its receptor, but diminished release of pituitary gonadotropins at ovulation and altered expression of ovarian markers, such as GDF9, important for follicular recruitment and survival [42]. So our results also confirmed the negative effects of leptin/lepR related obesity on reproduction.

In view of the local effects of leptin on ovary, although estradiol production is mainly regulated by FSH acting via the cAMP-dependent protein kinase (PKA) pathway in ovary granulosa cells [43, 44], lepR mediated JAK2-STAT3/STAT5 signaling pathway in local ovary was worthy of being determined. Leptin has been shown to primarily activate the JAK2-STAT3/STAT5 signaling pathway through three tyrosine sites on long isoform leptin receptor [45–47]. The long isoform LepR, which contains an intact intracellular domain with necessary protein motifs, can activate the JAK2, further stimulating the phosphorylation of multiple residues (Tyrosine 985, Tyrosine 1138, and Tyrosine 1077) on the intracellular domain of LEPR [48]. Phosphorylation of tyrosine residue 1138 mainly recruits STAT3 and it has been reported that hypothalamic leptin control to reproduction is regulated by signals independent of STAT3 signaling [49]. Meanwhile, Tyrosine 1077, the major phosphorylation site mediating leptin’s effects on reproduction, promotes the recruitment and transcriptional activation of STAT5 [50] and is required for ongoing appropriate function of the female reproductive function [51]. As expected, we found that in 12-week-old ovaries, phosphorylation of JAK2, STAT3 and STAT5 changed in Y123F ovaries, but no significant differences in 4-week-old ovaries between two genotypes.

Previously, Ding*,* et al. found that sileptin treatment decreased the secretion of E2 and the cell proliferation, but increased the secretion of progesterone and cell apoptosis in human ovarian granulosa cells in vitro. They confirmed that impaired activation of lepR mediating JAK2/STAT3 signal pathway contributed to ovarian granulosa cell apoptosis [52]. Therefore, our results indicated that LepR was involved in adult ovary steroidogenesis by mediating JAK2-STAT3/STAT5 signaling pathway in local ovaries.

## Conclusion

In conclusion, lepR tyrosine site mutations of Y123F mice not only decreased gonadotropin levels through the central nervous system, but also impaired steroidogenic enzymes expression of local ovary through JAK2-STAT3/STAT5 signaling pathways. Both effects led to abnormal sex hormones levels and infertility of Y123F mice. Owing to the local ovarian factors, exogenous hFSH supplement just partly restored the defects in serum E2 levels and ovary steroidogenic enzymes expression. Since obesity and obesity-related infertility/subfertility are growing problems in human beings, our finding that direct effects of leptin and lepR on ovary were involved in infertility of Y123F mice, suggested a new viewpoint on the relationship between obesity and reproduction. Accordingly, clinicians can try to improve their ovarian hormone synthesis function in obese patients with impaired fertility via supplement exogenous hFSH or hMG. Meanwhile, the founding that is, the feedback of gonadotropin action of Y123F mouse still existed, provides animal experimental basis for follow-up therapy with assisted reproductive technology on obesity-related infertility.

## Abbreviations

CYP11A1: P450scc, cholesterol side-chain cleavage enzyme
CYP19A1: Aromatase
E2: Estradiol
ELISA: Enzyme-linked immunosorbent assay
F: Phenylalanine
FSH: Follicle stimulating hormone
FSHR: Follicle stimulating hormone receptor
HPG: Hypothalamic-pituitary-gonadal
HSD17B7: Hydroxysteroid Dehydrogenase 17B7
IHC: Immunohistochemistry
JAK2: Janus kinase 2
LepR: Leptin receptor
LH: Luteinizing hormone
P: Progesterone
QPCR: Real-time quantitative PCR
STAR: Steroidogenic acute regulatory protein
STAT3: Signal transducer and activator of transcription 3
STAT5: Signal transducer and activator of transcription 5
T: Testosterone
WB: Western blot
WT: Wild type
Y: Tyrosine
Y123F: LepR tyrosine site mutation mice
